# STITCH 4: integration of protein–chemical interactions with user data

**DOI:** 10.1093/nar/gkt1207

**Published:** 2013-11-28

**Authors:** Michael Kuhn, Damian Szklarczyk, Sune Pletscher-Frankild, Thomas H. Blicher, Christian von Mering, Lars J. Jensen, Peer Bork

**Affiliations:** ^1^Biotechnology Center, TU Dresden, 01062 Dresden, Germany, ^2^Institute of Molecular Life Sciences, University of Zurich and Swiss Institute of Bioinformatics, Winterthurerstrasse 190, 8057 Zurich, Switzerland, ^3^Novo Nordisk Foundation Center for Protein Research, Faculty of Health Sciences, University of Copenhagen, 2200 Copenhagen N, Denmark, ^4^European Molecular Biology Laboratory, Meyerhofstrasse 1, 69117 Heidelberg, Germany and ^5^Max-Delbrück-Centre for Molecular Medicine, Robert-Rössle-Strasse 10, 13092 Berlin, Germany

## Abstract

STITCH is a database of protein–chemical interactions that integrates many sources of experimental and manually curated evidence with text-mining information and interaction predictions. Available at http://stitch.embl.de, the resulting interaction network includes 390 000 chemicals and 3.6 million proteins from 1133 organisms. Compared with the previous version, the number of high-confidence protein–chemical interactions in human has increased by 45%, to 367 000. In this version, we added features for users to upload their own data to STITCH in the form of internal identifiers, chemical structures or quantitative data. For example, a user can now upload a spreadsheet with screening hits to easily check which interactions are already known. To increase the coverage of STITCH, we expanded the text mining to include full-text articles and added a prediction method based on chemical structures. We further changed our scheme for transferring interactions between species to rely on orthology rather than protein similarity. This improves the performance within protein families, where scores are now transferred only to orthologous proteins, but not to paralogous proteins. STITCH can be accessed with a web-interface, an API and downloadable files.

## INTRODUCTION

Protein–chemical interactions are essential for any biological system; for example, they drive the metabolism of the cell or initiate many signaling cascades and most pharmaceutical interventions. A large collection of such interactions can, therefore, be used to study a variety of cellular functions and the impact of drug treatment on the cell. For such research, it is important to have, as complete as possible, data on protein–chemical interactions. By treating proteins and chemicals as nodes of a graph, which are linked by edges if they have been found to interact (1), we can adopt a network view that enables us to integrate many different sources. The concept of STITCH (‘search tool for interacting chemicals’) was from the beginning to combine sources of protein–chemical interactions from experimental databases, pathway databases, drug–target databases, text mining and drug–target predictions into a unified network (2–4). This network abstracts the complexity of the underlying data sources, making large-scale studies possible. At the same time, links to the original sources are retained, making it possible to trace the provenance of the data. The underlying STITCH database can be accessed in multiple ways: via an intuitive web interface, via download files (for large-scale analysis) and via an API (enabling automated access on a small to medium scale). Here, we present recent improvements to the database and user interface of STITCH. Already in the previous versions, it has been possible to query STITCH using protein or chemical names, InChIKeys and SMILES strings. New in this version is the possibility to upload spreadsheets with chemical descriptors and experimental data that can be directly added to the network, as described later in text. We also for the first time use the evidence transfer algorithm described for the STRING 9.1 database (5) to improve the performance for protein families.

**Figure 1.**
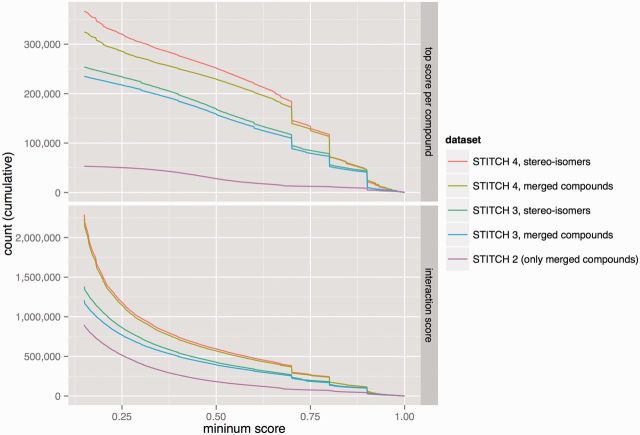
Cumulative distribution of scores. For each confidence score threshold, the plot shows the number of chemicals (top) and protein–chemical interactions (bottom) that have at least this confidence score in the human protein–chemical network. For example, there are 172 000 chemicals with a high-confidence interaction (score at least 0.7). As there are many interactions with low confidence scores, we use a minimum score threshold of 0.15. Steps in the data correspond to large numbers of compounds that have the same maximum score in manually curated databases or the ChEMBL database (with different confidence levels).

Compared with STITCH 3, we use the same underlying set of proteins, containing 1133 species. We updated the set of chemicals (6), and find interactions with 390 000 distinct chemicals. In human, high-confidence interactions for 172 000 compounds are available in STITCH 4 (Figure 1), compared with 110 000 in STITCH 3 (4). In total, the human protein–chemical interaction network contains 2.2 million interactions (Figure 1). Applying different confidence thresholds, 570 000 interactions are of medium confidence (score cutoff 0.5) and 367 000 interactions are of high confidence (cutoff 0.7).

## SOURCES OF INTERACTIONS

Protein–chemical interactions are presented in four different channels: experiments, databases, text mining and predicted interactions. We import the following sources of experimental information: ChEMBL [interactions with reported K_i_ or IC_50_ (7)], PDSP K_i_ Database (8), PDB (9) and—new to STITCH—data from two large-scale studies on kinase–ligand interactions (10,11). From the latter studies, we extracted 74 291 interactions between 229 compounds and 414 human kinases. We converted the reported residual kinase activities (10) and kinase affinities (11) to probabilistic scores, which gave rise to 14 187, 9431 and 5977 interactions of at least low, medium and high confidence, respectively. The second channel is made up of manually curated drug–target databases: DrugBank (12), GLIDA (13), Matador (14), TTD (15) and CTD (16); and pathway databases: KEGG (17), NCI/Nature Pathway Interaction Database (18), Reactome (19) and BioCyc (20)*.*

## PREDICTION OF INTERACTIONS

STITCH contains verified interactions (from the sources listed earlier in text) and predicted interactions, based on text mining and other prediction methods. In the text-mining channels, interactions were extracted from the literature using both co-occurrence text mining and Natural Language Processing (21,22). For the first time for STITCH, we not only use data from MEDLINE abstracts and OMIM (23) but also from full-text articles freely available from PubMed Central or publishers’ Web sites.

In previous versions, we have used medical subject headings (MeSH) terms in text mining and when importing external databases. These terms allowed us to expand concepts like ‘alpha adrenergic receptors’ to individual proteins. We used to map MeSH terms to proteins using a combination of automatic and manual approaches, which led to errors in some cases. Furthermore, the mapping was only valid for human proteins. We have, therefore, started to use terms from the Gene Ontology [GO terms, (24)] to define groups of proteins. We excluded GO annotations based on mutant phenotypes (IMP) and electronic annotations (IEA). We then checked the coverage of GO annotations for all species in STITCH. We only mapped GO terms to proteins for species where at least 10% of the proteins have been annotated, namely *Drosophila melanogaster*, *Escherichia coli*, *Homo sapiens*, *Mus musculus*, *Saccharomyces cerevisiae* and *Schizosaccharomyces pombe*.

As the coverage of synonyms is lower than for MeSH terms, we manually added additional synonyms to GO terms to increase the text-mining sensitivity. As one GO term corresponds to multiple proteins, the resulting confidence score for the individual protein–chemical interactions should be down-weighted compared with interactions that are directly associated with a single protein. We, therefore, determined a correction factor through benchmarking (as a function of the number of member proteins in the GO term). For each channel, we looked at the GO terms that are interacting with chemicals. We then checked if the member proteins that are part of the GO terms are in turn interacting with chemicals. For each of these chemicals, we determined the fraction of member proteins that are interacting. For example, if a drug was known to bind two of the three α2-adrenergic receptors, it was added as a data point (x = 3, y = 2/3) to the benchmark data. The data points were then fitted for each channel by the following function:

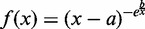

For larger groups, the function approaches *x^−^^1^* (i.e. interacting with one protein is not predictive for the other proteins).

In this version of STITCH, we introduced a fourth channel, namely predicted protein–chemical interactions based on chemical structure. Countless articles on the prediction of drug–target interactions have been published in the last years [e.g. (25–27), reviewed in (28)]. In many cases, however, the actual predictions are not available. We, therefore, implemented a relatively simple and transparent prediction scheme based on Random Forests (29,30): for each target for which >100 binding partners are known from the ChEMBL database, we attempted to make a prediction. To avoid biases, we first excluded highly similar chemicals, enforcing a maximum Tanimoto similarity of 0.9 (using Algorithm 2 described by Hobohm) (31) using 2D chemical fingerprints calculated with the chemistry development kit (32,33). We then added ten times as many random chemicals as non-binders to the training set and used the fingerprints as predictors for all compounds. Using 10-fold cross-validation, we assessed how predictive the model is (by calculating the Pearson correlation coefficient between the training data and the cross-validation results). We used the correlation as a correction factor to decrease the confidence score of the predicted interactions, which were predicted for all compounds occurring in the ChEMBL database. We repeated this procedure three times for each compound and used the median predicted score, to decrease the effect of the random negative set. As interactions were predicted from the experimental channel, the predictions and experimental channels are not independent of each other. To compute the combined score (which is shown on the network), we therefore took the highest of either score, instead of combining the scores in a Bayesian fashion as it is done for the other channels. In total, predictions were made for 767 proteins across 15 species. The median correlation between the training data and the cross-validation prediction was 0.90.

Links between compounds were also extracted from the aforementioned sources, if possible. (e.g. chemical reactions from pathway databases or co-mentioned chemicals from text mining.) We also predicted shared mechanisms of action from MeSH pharmacological actions, the Connectivity Map using the DIPS method (34), which tests for similar changes in gene expression on compound treatment, and from screening data from the Developmental Therapeutics Program NCI/NIH (35). The latter screening data replaces our previous analysis of the NCI60 panel. We considered only the 70 of 115 cell lines against which >10 000 compounds have been screened and centered the negative logarithm of GI50 values with respect to both compounds and cell lines. For the 47 692 compounds in the data set, we calculated all-against-all covariance across cell lines and converted these to probabilistic scores. This resulted in 114 072, 24 889 and 6890 pairs of compounds of at least low, medium and high confidence, respectively.

To account for the fact that many interactions are determined in model species, we transfer interactions between species. Previously, the sequence similarity between two proteins was used to determine the confidence in the transferred score. This had the disadvantage that when transferring evidence from a selective binder (e.g. inhibiting only one subtype of a receptor), all subtypes of the receptor in the target species would receive a similar score. In the new scheme, only the orthologous protein receives the evidence from the specific compound.

## INTEGRATION WITH USER DATA

**Figure 2.**
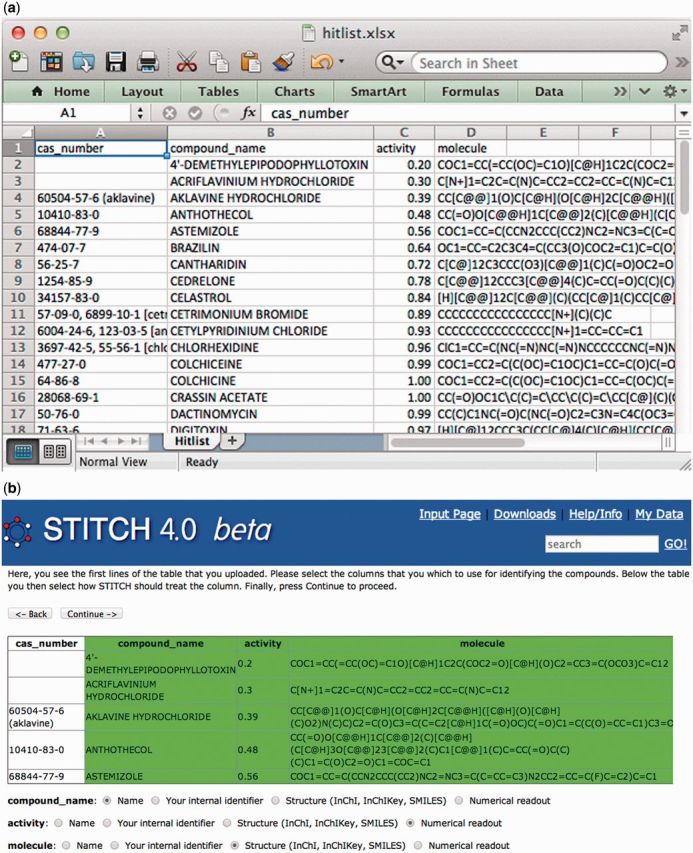
Data upload. The user can use the batch import form to upload a spreadsheet, e.g. from Microsoft Excel (**a**). STITCH will then show the first five rows of the spreadsheet and ask the user to identify columns that contain the name, chemical structure or a numerical readout (**b**). Selected columns are highlighted in green. STITCH uses heuristics to suggest which kind of information the columns contain, e.g. by identifying SMILES strings as structural descriptors.

**Figure 3.**
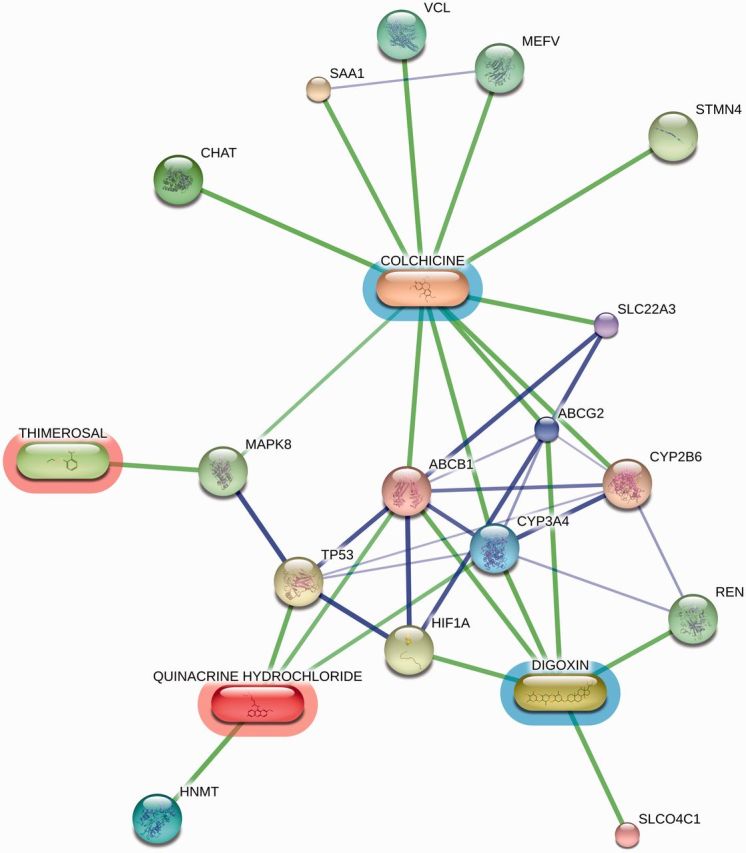
User data and the STITCH network. For four compounds that are part of the example data set from Figure 2, interacting proteins are shown. The numerical readout has been converted to a color on a red–blue gradient. Instead of the normal chemical names used by STITCH, the full names provided in the data set are used, enabling the user to easily recognize the studied chemicals.

Users can now upload a spreadsheet (e.g. in Microsoft Excel format) with experimental data to STITCH using the ‘batch import’ functionality (Figure 2). For each compound, the spreadsheet may contain: the name of the compound, the chemical structure (as SMILES string, InChI or InChIKey), an internal identifier and a readout value. STITCH uses the name and chemical structure to find the compound in the STITCH database. The name provided by the user can then be shown in the interaction network, and the downloadable files contain both the name and the user’s internal identifier (if provided). The readout value may be a numerical value, e.g. the activity of a compound in a screen. The user can then select a palette from the ColorBrewer2 color schemes (36). The palette is used to convert the numerical value into a color, which is then used to highlight the compounds in the network with a colored halo (Figure 3). It is also possible to directly specify colors (in standard hexadecimal notation).

## USE CASES

The majority of users access STITCH via the web interface, where networks can be retrieved using single or multiple names of proteins or chemicals. Furthermore, users can query STITCH with protein sequences and chemical structures (in the form of SMILES strings). The networks can then be explored interactively or saved in different formats, including publication-quality images. Proteins and chemicals can be clustered in the interactive network viewer and enriched GO terms among the proteins can be computed (5,37). The set of all interactions is also available for download under Creative Commons licenses (with separate commercial licensing for a subset). In this way, STITCH can be used to drive large-scale studies. Many research groups have already used STITCH 3 in this way; a few examples illustrating different utilities follow: STITCH has been used to determine which proteins cause side effects during drug treatment (38,39) by combining the STITCH network with data from a side effect database (40). The database has also been instrumental for the identification of druggable proteins to predict polypharmacological treatment of diseases on the basis of network topology features (41). For a method that predicts drug targets based on chemogenetic assays in yeast, STITCH has been chosen as a benchmark set (42). Lastly, STITCH has also been integrated into other tools, for example ResponseNet2.0 and QuantMap (43,44).

## FUNDING

Deutsche Forschungsgemeinschaft [DFG KU 2796/2-1 to M.K.]; Novo Nordisk Foundation Center for Protein Research. Funding for open access charge: European Molecular Biology Laboratory.

*Conflict of interest statement*. None declared.
